# Performance of the Visual Analogue Scale of Happiness and of the Cornell Scale for Depression in Dementia in the Tremembé Epidemiological Study, Brazil

**DOI:** 10.1590/S1980-57642014DN84000014

**Published:** 2014

**Authors:** Karolina G. César, Sonia M.D. Brucki, Leonel T. Takada, Luiz Fernando C. Nascimento, Camila M.S. Gomes, Milena C.S. Almeida, Maira O. Oliveira, Fábio H.G. Porto, Mirna L.H. Senaha, Valéria S. Bahia, Thaís Bento L. Silva, Jéssica N. Ianof, Lívia Spíndola, Magali T. Schmidt, Mário S. Jorge, Patrícia H.F. Vale, Mário A. Cecchini, Luciana Cassimiro, Roger T. Soares, Márcia R. Gonçalves, Jerusa Smid, Claudia S. Porto, Maria Teresa Carthery-Goulart, Mônica S. Yassuda, Letícia L. Mansur, Ricardo Nitrini

**Affiliations:** 1 MD, PhD. Cognitive and Behavioral Neurology Unit, Department of Neurology, University of São Paulo, Brazil; 2 Professor at the University of Taubaté, Brazil; 3 Tremembé Epidemiologic Study (TES) Group; 4 MD, PhD, Full Professor. Professor of Neurology, University of São Paulo Medical School. Cognitive and Behavioral Neurology Unit, Department of Neurology, University of São Paulo, Brazil.

**Keywords:** depression, elderly, prevalence, Cornell Scale for Depression, Visual Analogue Scale of Happiness

## Abstract

Depression is a major growing public health problem. Many population studies have found a significant relationship between depression and the presence of cognitive disorders.

**Objective:**

To establish the correlation between the Visual Analogue Scale of Happiness and the Cornell Scale for Depression in Dementia in the population aged 60 years or over in the city of Tremembé, state of São Paulo, Brazil.

**Methods:**

An epidemiological survey involving home visits was carried out in the city of Tremembé. The sample was randomly selected by drawing 20% of the population aged 60 years or older from each of the city's census sectors. In this single-phase study, the assessment included clinical history, physical and neurological examination, cognitive evaluation, and application of both the Cornell Scale and the Analogue Scale of Happiness for psychiatric symptoms. The presence of depressive symptoms was defined as scores greater than or equal to 8 points on the Cornell Scale.

**Results:**

A total of 623 subjects were evaluated and of these 251 (40.3%) had clinically significant depressive symptoms on the Cornell Scale, with a significant association with female gender (p<0.001) and with lower education (p=0.012). One hundred and thirty-six participants (21.8%) chose the unhappiness faces, with a significant association with age (p<0.001), female gender (p=0.020) and low socioeconomic status (p=0.012). Although there was a statistically significant association on the correlation test, the correlation was not high (rho=0.47).

**Conclusion:**

The prevalence of depressive symptoms was high in this sample and the Visual Analogue Scale of Happiness and Cornell Scale for Depression in Dementia should not be used as similar alternatives for evaluating the presence of depressive symptoms, at least in populations with low educational level.

## Abstract (pt)

A depressão é um problema importante e crescente de saúde pública. É muito comum ser encontrada uma relação significativa entre depressão e a presença de distúrbios cognitivos nos estudos populacionais.

**Objetivo:**

Estabelecer a correlação entre a Escala Analógica Visual de Felicidade e a Escala Cornell de Depressão em Demência na população de 60 anos ou mais da cidade de Tremembé, estado de São Paulo, Brasil.

**Métodos:**

Estudo epidemiológico no qual foram realizadas visitas domiciliares na cidade de Tremembé. A amostra foi aleatória, através do sorteio de 20% da população acima de 60 anos de cada setor censitário do município. Este estudo foi de fase única, tendo sido realizada anamnese, exames físico e neurológico, avaliação cognitiva e aplicação de escalas Cornell de Depressão em Demência e Escala Analógica de Felicidade para verificar a presença de sintomas depressivos. Foi adotado como critério da presença de sintomas depressivos, pontuação maior ou igual a 8 na escala de Cornell.

**Resultados:**

Foram avaliadas 623 pessoas e destas 251 (40,3%) apresentaram sintomas depressivos significativos clinicamente na escala de Cornell, com associação significativa com gênero feminino (p<0,001) e com a baixa escolaridade (p=0,012). Cento e trinta e seis participantes (21,8%) apontaram para faces de infelicidade, com associação significativa com idade (p<0,001), com gênero feminino (p=0,020) e com baixo nível socioeconômico (p=0,012). Embora tenha havido significância estatística no teste de correlação, a correlação entre as duas escalas analisadas não foi alta (rho=0,47).

**Conclusão:**

A prevalência de sintomas depressivos foi elevada nesta amostra e a Escala Analógica de Felicidade e a Escala Cornell de Depressão em Demência não devem ser utilizadas como alternativas similares para avaliar a presença de sintomas depressivos, pelo menos em populações com baixa escolaridade.

## INTRODUCTION

Depression is a major and growing public health problem and possibly the leading cause of mental disability.^1^ It is very common in the elderly population and many population studies have found a significant relationship between depression or depressive symptoms and the presence of cognitive disorders.^2-4^

Several instruments can be used to detect and measure level of depression, two of which stand out for their practical application: the Cornell Scale^5^ and the Visual Analogue Scale of Happiness (VASH).^6^ The Cornell Scale, despite having been initially developed for the diagnosis and monitoring of depression in patients with dementia, is also a validated instrument for use in both demented and non-demented geriatric subjects.^7^ It is also slightly more comprehensive than the geriatric depression scale,^8,9^ by covering issues related to anxiety, behavioral and sleep changes. The VASH contains six faces expressing from great happiness to deep sadness or unhappiness, and the participant need only indicate which face best identifies their mood.^6^

The aim of this study was to establish the correlation between the VASH and the Cornell Depression Scale in an epidemiological study conducted to diagnose cognitive disorders in the population aged 60 years or over in the city of Tremembé, state of São Paulo, Brazil.

## METHODS

This study was an epidemiological study in which home visits were carried out in the city of Tremembé, located in the State of São Paulo, about 140 km from the State capital. According to the population census conducted in 2011 by the Brazilian Institute of Geography and Statistics (IBGE), Tremembé had a population of 40,751 inhabitants, of whom 3,690 were aged 60 years or more (185 of whom lived in rural areas).^10^ This study was approved by the University of São Paulo Research Ethics Committee (protocol 0378/09).

**Sampling.** The initial parameters of the sample were estimated for a study on the prevalence of cognitive impairment with and without dementia, which was the primary objective of this study.^6^ Twenty percent of the households with individuals aged 60 years or over were randomly selected from each of the municipality's census sectors to obtain a homogeneous representation of all regions and districts as well as different socioeconomic levels. Seven hundred and thirty-eight individuals aged 60 years or more were randomly selected from both urban and rural areas.

Following selection, letters were sent by mail inviting subjects to participate in the study. Subsequently, a community agent visited the homes and scheduled a home visit. The subjects or legal guardians were fully informed about the study and signed a consent form. Only one individual was included from each selected household. Individuals institutionalized in either of the city's nursing homes were included in the study following randomization, but only individuals who were randomly selected in their respective census sector were examined at these institutions.

**Exclusion criteria.** The study excluded only those who did not have informants to help answer the questionnaires. When the elderly drawn refused to participate, we invited the nearest neighbor aged 60 years or more to participate, to minimize sample loss and try to maintain the percentage of subjects sampled from each sector. The three city's prisons sectors were excluded from the study and another nine sectors were also excluded because they contained no residents aged 60 years or over.

**Assessment.** A cross-sectional survey was conducted in which history taking, physical and neurological examination, cognitive assessment, psychiatric evaluation and functional activity questionnaires were carried out in a single-phase visit.^11^

Two previously cited scales were employed for the evaluation of psychiatric symptoms: the Cornell Depression Scale^5,12^ and the Patient Health Questionnaire (PHQ) from the Primary Care Evaluation of Mental Disorders (PRIME-MD) which included the VASH.^6,13^

The diagnostic criterion adopted for clinically significant depressive symptoms was a score greater than or equal to 8 points on the Cornell scale.^7^ For the VASH, subjects that chose the faces Little Unhappy, Unhappy or Very Unhappy were considered as presenting depressive symptoms.

**Statistical analysis.** Statistical analyses were performed using the SPSS (Statistical Package for the Social Sciences) version 17.0 software. The degree of association between the Cornell Scale and the Visual Analogue Scale of Happiness, and age, level of education, gender and socioeconomic status, was determined by Pearson's Chi-square test between crossed variables. The degree of correlation between the two scales was determined by Spearman's test and the evaluation of agreement between both scales by McNemar's test.

## RESULTS

Twenty percent of the population over 60 years of age was randomly selected, which corresponded to 738 households. Of these, 630 subjects agreed to participate although seven were in advanced stages of dementia and could not answer the Cornell Scale and VASH. This gave a final study sample of 623 participants.

Two hundred and fifty-one participants (40.3%) who answered the Cornell scale were diagnosed with clinically significant depressive symptoms (Cornell ≥8 points). One hundred and thirty-six participants (21.8%) chose a face with some degree of unhappiness on the VASH (Table 1).

**Table 1:** Association between no. of depression cases detected on the Cornell scale and on the Visual Analogue Scale of Happiness.

		Cornell		Total	p Value*
		Without depression	With depression		
V.A.S.H.	Very happy	133	35	168	< 0.001
	Happy	119	31	150	
	Neutral	82	87	169	
	Little Unhappy	25	51	76	
	Unhappy	6	19	25	
	Very unhappy	7	28	35	
Total		372	251	623	

V.A.S.H.: Visual Analogue Scale of Happiness;

* Spearman’s Correlation; without depression < 8 and with depression ≥8 points on the Cornell Scale.

**Figure 1:**
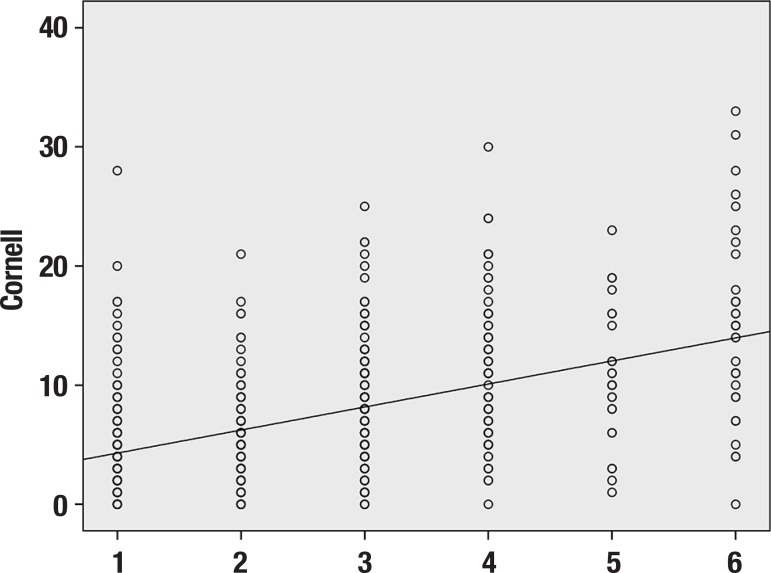
Correlation between scores on the Cornell scale and on the Visual Analogue Scale of Happiness scale.

There was a significant difference between the two scales (p<0.001) (Table 1) where Spearman's correlation coefficient was low (0.47) and then showed disagreement. Therefore, these two scales do not measure exactly the same variable. Analysis of agreement between diagnoses obtained using both scales (McNemar's test) showed the same significant disagreement (p<0.001). Thus, a high score found on the Cornell scale did not necessarily coincide with an unhappy face on Visual Analogue Scale of Happiness, as shown in Figure 1.

Regarding the Cornell scale, female gender (p<0.001) as well as lower education (p=0.012) showed a significant correlation with the presence of the diagnosis of clinically significant depressive symptoms (Table 2). The association with socioeconomic level almost reached significance (p=0.053).

**Table 2:** Cornell scale by age, gender, education and socioeconomic level.

Variables		Cornell Scale			
		Without depression (%) N= 372	With depression (%) N= 251	p Value*	Total (%) N=623
Age groups				0.176	
	60-64 years	82 (22.0)	70 (27.9)		152 (24.4)
	65-69 years	93 (25.0)	50 (23.5)		152 (24.4)
	70-74 years	75 (20.2)	41 (16.3)		116 (18.6)
	75-79 years	64 (17.2)	34 (13.5)		98 (15.7)
	80-84 years	36 (9.7)	26 (10.4)		62 (10.0)
	≥85 years	22 (5.9)	21 (8.4)		43 (6.9)
Gender				<0.001	
	Male	166 (44.6)	63 (25.1)		229 (36.8)
	Female	206 (55.4)	188 (74.9)		394 (63.2)
Years of education				0.012	
	Illiterate	44 (11.8)	42 (16.7)		86 (13.8)
	1-4 years	200 (53.8)	143 (57.0)		343 (55.1)
	5-8 years	46 (12.4)	37 (14.7)		83 (13.3)
	9-11 years	36 (9.7)	14 (5.6)		50 (8.0)
	≥12 years	46 (12.4)	15 (24.6)		61 (9.8)
Socioeconomic level**				0.053	
	A	21 (5.7)	4 (1.6)		25 (4.0)
	B	118 (31.7)	22 (8.8)		182 (29.1)
	C	178 (47.9)	134 (53.4)		312 (50.1)
	D	55 (14.8)	48 (19.1)		103 (16.6)
	E	0 (0.0)	1 (0.4)		1 (0.2)

* Pearson’s χ^2^ test.

** ABIPEME: Brazilian Association of Market Research (ranging from A [highest] to E [lowest]). Without depression < 8, and with depression ≥ 8 points, on the Cornell Scale.

In relation to the VASH, there was no significant association of education with unhappy mood but there was an association with age (p<0.001), female gender (p=0.020) and low socioeconomic status (p=0.007) (Table 3) was detected.

**Table 3:** Visual Analogue Scale of Happiness by age, gender, education and socioeconomic level.

Variables		Visual Analogue Scale of Happiness							
		Very Happy (%) N=168	Happy (%) N=150	Neutral (%) N=169	Little Unhappy (%) N=76	Unhappy (%) N=25	Very Unhappy (%) N=35	p Value*	Total (%) N=623
Age groups								<0.001	
	60-64 years	37 (22.0)	29 (19.3)	49 (29.0)	21 (27.6)	10 (40.0)	6 (17.1)		152 (24.4)
	65-69 years	43 (25.6)	43 (28.7)	37 (21.9)	18 (23.7)	3 (12.0)	8 (22.9)		152 (24.4)
	70-74 years	20 (11.9)	40 (26.7)	33 (19.5)	13 (17.1)	3 (12.0)	7 (20.0)		116 (18.6)
	75-79 years	40 (23.8)	18 (12.0)	18 (10.7)	10 (13.2)	4 (16.0)	8 (22.9)		98 (15.7)
	80-84 years	20 (11.9)	14 (9.3)	18 (10.7)	3 (3.9)	3 (12.0)	4 (11.4)		62 (10.0)
	≥85 years	8 (4.8)	6 (4.0)	14 (8.3)	11 (14.4)	2 (8.0)	2 (5.7)		43 (6.9)
Gender								0.020	
	Male	58 (34.5)	66 (44.0)	67 (39.6)	23 (30.3)	10 (40.0)	5 (14.3)		229 (36.8)
	Female	110 (65.5)	84 (56.0)	102 (60.4)	53 (69.7)	15 (60.0)	30 (85.7)		394 (63.2)
Years of education								0.116	
	Illiterate	24 (14.3)	14 (9.3)	23 (13.6)	14 (18.4)	4 (16.0)	7 (20.0)		86 (13.8)
	1-4 years	95 (56.5)	82 (54.7)	83 (49.1)	45 (59.2)	17 (68.0)	21 (60.0)		343 (55.1)
	5-8 years	21 (12.5)	16 (10.7)	27 (16.0)	12 (15.8)	2 (8.0)	5 (14.3)		83 (13.3)
	9-11 years	10 (6.0)	17 (11.3)	16 (9.5)	4 (5.3)	1 (4.0)	2 (5.7)		50 (8.0)
	≥ 12 years	18 (10.7)	21 (14.0)	20 (11.8)	1 (1.3)	1 (4.0)	0 (0.0)		61 (9.8)
Socioeconomic level**								0.007	
	A	4 (2.4)	11 (7.4)	10 (5.9)	0 (0.0)	0 (0.0)	0 (0.0)		25 (4.0)
	B	59 (35.1)	50 (33.4)	50 (29.6)	17 (22.4)	3 (12.0)	3 (8.6)		182 (29.1)
	C	83 (49.4)	65 (43.3)	78 (46.2)	46 (60.5)	18 (72.0)	22 (62.8)		312 (50.1)
	D	22 (13.1)	24 (16.0)	31 (18.3)	13 (17.1)	4 (16.0)	9 (25.7)		103 (16.6)
	E	0 (0.0)	0 (0.0)	0 (0.0)	0 (0.0)	0 (0.0)	1 (2.9)		1 (0.2)

* Pearson’s χ^2^ test.

** ABIPEME: Brazilian Association of Market Research (ranging from A [highest] to E [lowest]).

## DISCUSSION

Depression appears in some studies as another risk factor for the onset of cognitive impairment, but it could be a prodrome or even be a possible cause of reversible dementia.^14^ The management of depressive symptoms is crucial in outpatient care of elderly since depression is a predictor of decline in functional abilities.

In previous reported studies, the combined prevalence of significant depressive symptoms and major depressive disorder in elderly Brazilians was 7% in São Paulo^15^ and depression in the general population was 16.1% in Porto Alegre.^16^ In the present study, we found a prevalence of 40.3% of clinically significant depressive symptoms using the Cornell Scale for Depression in Dementia, while the prevalence of unhappiness was 21.8% when the VASH was employed.

Our data are insufficient to evaluate which of these two scales is most appropriate to detect depressive symptoms in population studies. However, the prevalence obtained with the VASH, where 21.8% of participants chose the face with some degree of unhappiness, was closer to the previously reported prevalence of depression among the elderly in Brazil.^14-16^ In the SABE study (Health, well-being and aging), the prevalence of depression in the city of São Paulo was 18.1% when the Geriatric Depression scale was used.^17^

The VASH is easier and much faster to use than the Cornell Scale questionnaire. The Cornell Scale detected depressive symptoms in 40.3% of the sample while the VASH showed depressive symptoms in 21.8%, a figure which may be closer to the actual rate of depression in the elderly of our community. However, this theory needs to be further tested.

In conclusion, the VASH and the Cornell Scale for Depression in Dementia should not be used as similar alternatives to evaluate the presence of depressive symptoms, at least in populations with low educational level. Further studies are needed to evaluate whether the VASH may be used in epidemiologic studies for the detection of depressive symptoms in poorly educated populations.
